# Prenatal Echo-Sonographic Parameters in Fetuses Wrapped with the Umbilical Cord in the Third Trimester of Pregnancy

**DOI:** 10.3390/jcm12196170

**Published:** 2023-09-24

**Authors:** Julia Murlewska, Oskar Sylwestrzak, Sławomir Witkowski, Maria Respondek-Liberska, Maciej Słodki, Iwona Strzelecka

**Affiliations:** 1 Department of Prenatal Cardiology, Polish Mother’s Memorial Hospital Research Institute, 93-338 Lodz, Poland; 2 Department of Diagnoses and Prevention of Fetal Malformations, Medical University of Lodz, 90-419 Lodz, Poland; 3 Institute of Health Science, The State School of Higher Professional Education, The State University of Applied Sciences in Plock, 09-402 Plock, Poland

**Keywords:** umbilical artery, Tei index, umbilical cord around fetal neck, fetal echo, normal heart anatomy, caesarean section

## Abstract

This study constitutes a description of prenatal echo-sonographic parameters in fetuses wrapped with the umbilical cord in the third trimester of pregnancy and demonstrates the practical importance of the umbilical cord collision. Echocardiographic examinations were performed within 6 months, and a group of patients in the third trimester with a mean age of 28.1 ± 0.79 weeks of gestation (*p* = 0.075) was distinguished. The group included 46 fetuses from single pregnancies with the umbilical cord around the fetal neck and 70 fetuses without the umbilical cord around the fetal neck. The course of the umbilical cord coiling around the fetal neck was recorded by color Doppler. We also conducted a follow-up with the newborns. In the study group, there was an elevated peak systolic velocity of the umbilical artery (UMB PSV) at a level of 44.17 cm/s vs. 38.90 cm/s in the control group (*p* = 0.004), and caesarean sections were significantly more frequent (54.5% vs. 31.4%). The persistence of the nuchal cord during delivery was observed in 37% of newborns in the study group, while the occurrence of umbilical wrapping during delivery was found in 18.6% of newborns in the control group (*p* = 0.027). In the studied cases, caesarean sections were 2.62 times more frequent (OR = 2.62), whereas nuchal cords during delivery were 2.57 times more often observed (OR = 2.57). Fetal umbilical cord wrapping in the third trimester of pregnancy does not have a significant hemodynamic impact; however, the UMB PSV might be slightly elevated in this group, and the frequency of umbilical cord collision during delivery and the need to perform a caesarean section at term seem to be more common.

## 1. Introduction

Wrapping of the umbilical cord around the fetal neck, called a nuchal cord, is a common phenomenon observed prenatally in all three trimesters of pregnancy, as well as during delivery. Fetal umbilical cord wrapping can be considered in terms of possible compression and its effects on umbilical circulation and myocardial function, which are determined with the use of the Tei indices for the fetal left and right ventricles. The nuchal cord may constitute a problem when the cord is tightly wrapped around the fetal neck [1,2]. The available scientific studies often refer to the possible adverse consequences of umbilical cord collision on the fetus in the final hours of pregnancy before birth, which contribute to the occurrence of cardiac decelerations, prolonged labor duration, or perinatal hypoxia. These perinatal abnormalities associated with the nuchal cord may, in turn, lead to deterioration of the umbilical cord blood acid-base balance parameters and Apgar scores. Moreover, increased neonatal needs for oxygen, resuscitation, and neonatal intensive care unit (NICU) admissions were observed in the newborns with the umbilical cord around their neck during labor. There are still many myths about the umbilical cord around the fetal neck. A lot of patients are concerned about the presence of the nuchal cord. A detailed ultrasound analysis of the umbilical cord is therefore needed [3,4]. The aim of our study was to compare a group of fetuses with the umbilical cord around the fetal neck and a group of fetuses without the umbilical cord around the fetal neck. We compared the maternal data, prenatal echo-sonographic parameters, and postnatal condition of newborns. Fetal echocardiographic examinations were performed according to a family’s history of heart defects or past obstetric complications.

## 2. Materials and Methods

The study included two groups of fetuses: a study group (n = 46 fetuses) with the umbilical cord around the fetal neck and a control group (n = 70) without the umbilical cord around the fetal neck, with a mean age of 28.1 ± 0.79 weeks of gestation. This was a cross-sectional study encompassing the period of 2019–2020. The quantitative differences in both groups (46 patients from the group with the umbilical cord collision versus 70 patients from the control group) resulted from the actual occurrence of the umbilical cord collisions in the study population. The echocardiographic examinations were performed at a reference center for prenatal cardiology in Lodz according to the criteria developed by the International Prenatal Cardiology Collaboration Group [2]. All measurements were taken by one experienced echocardiographer (JM). The criteria for inclusion in the study were as follows: single pregnancy with a determined gestational age based on the last menstrual period, sonographic biometry, availability of completed maternal medical records, and known postnatal condition of newborns. The echocardiographic measurements were performed in the third trimester of pregnancy. Study groups were matched in terms.

Exclusions from our study included patients with an unknown date of the last menstrual period, irregular periods before pregnancy, congenital heart defects, congenital extra-cardiac defects of the fetus, genetic abnormalities, intrauterine growth restriction of the fetus, multiple pregnancy, maternal chronic hypertension, preeclampsia, pregestational or gestational diabetes, and any other chronic maternal diseases.

**Figure 1:**
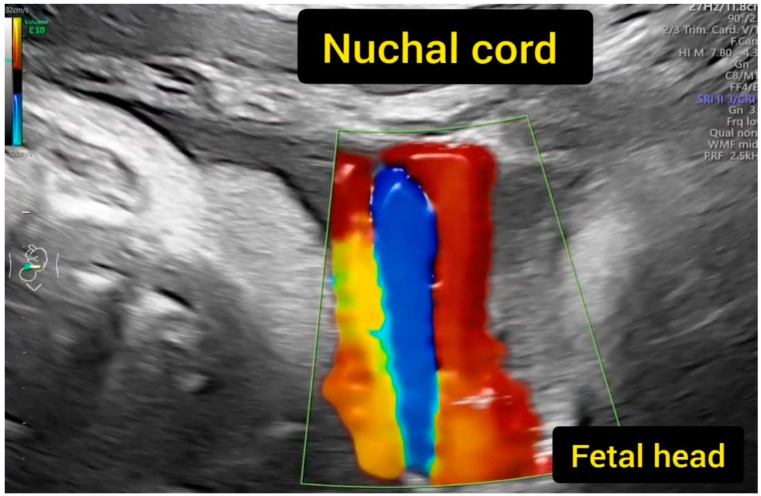
Status evaluation of the umbilical cord around the fetal neck with color Doppler.

**Figure 2:**
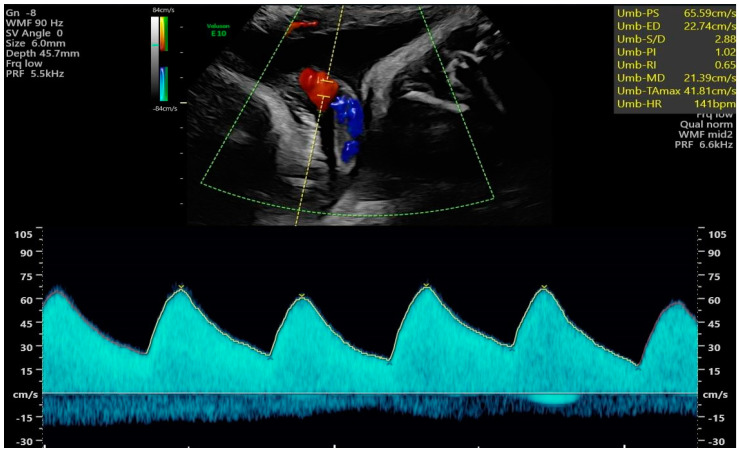
Evaluation of the UMB PSV [cm/s] in a fetus wrapped with the umbilical cord.

We used the GE Voluson E10 BT18 ultrasound machine equipped with the fetal echo program and transabdominal RAB6-D and C2-9-D probes. The Tei index is the quotient of the sum of the durations of isovolumetric contraction time (IVCT) and isovolumetric relaxation time (IVRT) divided by the ejection time (ET). In order to acquire the best repeatable values of the Tei index, we obtained images of the fetal four-chamber views, followed by valve clicks to identify time periods of the cardiac cycle [1,2,5]. The status of the umbilical cord around the fetal neck was evaluated with color Doppler in every patient (Figure 1, Clip 1, Figure 2); however, this was not included in the medical report from the echo examination. The other analyzed echo-sonographic parameters included cardiothoracic area ratio (CTAR), fetal heart rate (FHR) [bpm], UMB PSV [cm/s], umbilical artery pulsatility index (UMB PI), amniotic fluid index (AFI), and estimated fetal weight (EFW). Larger for gestational age (LGA) fetuses were found above the 90th percentile and smaller for gestational age (SGA) fetuses were found below the tenth percentile for gestational age, while appropriate for gestational age (AGA) fetuses had weights estimated between the 10th and 90th percentile for gestational age, according to Hadlock’s formula [6]. The data about the maternal urogenital tract infection, newborn infections, week of delivery, newborn weight, method of delivery, umbilical cord wrapping around the fetal neck during delivery, newborn condition (Apgar scores in 1, 5 min of life), and the occurrence of jaundice associated with elevated blood bilirubin levels in a newborn requiring phototherapy and hospital stay [days] were also analyzed. The data were retrieved from hospital records.

We confirm that all experimental protocols were approved by the Bioethical Commission at the Medical University of Łódź (RNN/286/20/KE, 17 April 2020), all methods were carried out in accordance with relevant guidelines and regulations, and informed consent was obtained from all the participants.

Statistical analyses were performed with the use of IBM SPSS Statistics 25.0. α = 0.05 was used as the significance level. In order to check which of the analyzed variables differentiated fetuses with the umbilical cord around the fetal neck (n = 46) from the study group and those without the umbilical cord around the fetal neck (n = 70) from the control group in the third trimester of pregnancy, the Pearson’s χ^2^ test or Fisher’s exact test were performed. The Student’s *t*-test was used for independent samples. In order to predict the occurrence of umbilical cord wrapping around the fetal neck, a ROC curve analysis was performed to determine the values of Tei for RV and Tei for LV.

## 3. Results

**Figure 3:**
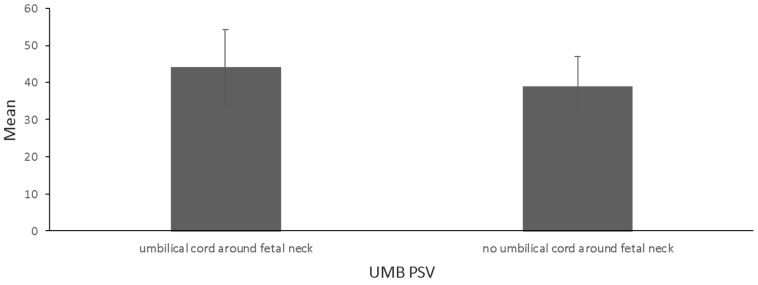
Mean and standard deviation for UMB PSV [cm/s] among fetuses in a study group (wrapped with the umbilical cord) and control group (44.17 vs. 38.90, *p* = 0.004), with a mean age of 28.1 ± 0.79 weeks of gestation.

In the study group, the UMB PSV parameter was higher than in fetuses in the control group (44.17 cm/s vs. 38.90 cm/s; *p* = 0.004) (Figure 3, Table 1). The strength of the effect on differences was moderate. With respect to other parameters, the differences turned out to be insignificant (Table 1).

**Figure 4:**
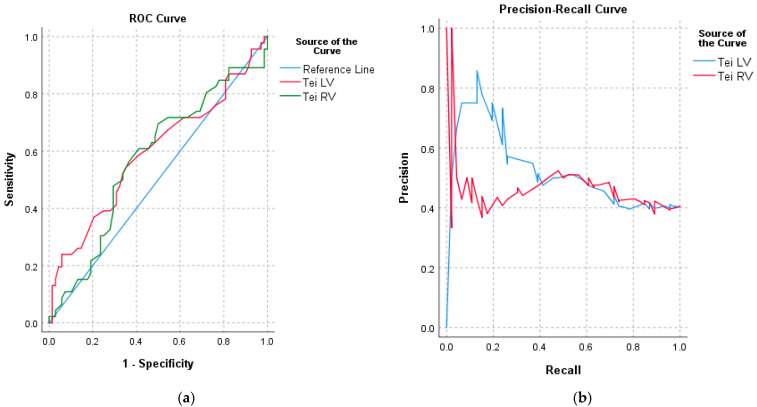
(**a**) ROC curve for RV Tei and LV Tei obtained in the third trimester of pregnancy for fetuses with the umbilical cord around the fetal neck—the study group. (**b**) Prediction accuracy of the umbilical cord around the fetal neck at the time of echo-sonographic examination in the third trimester based on the obtained LV Tei and RV Tei values.

For RV Tei, the AUC (area under curve) value was 0.57 (*p* = 0.196), while for LV Tei, the AUC was 0.59 (*p* = 0.095), which indicates that the probability of the umbilical cord wrapping around the fetal neck based on RV Tei and LV Tei parameter values is accidental. Therefore, a cut-off threshold cannot be determined from the available data (Figure 4a,b).

The analysis showed that in the study group (fetuses wrapped with the umbilical cord during echo-sonographic examination in the third trimester of pregnancy), caesarean sections were significantly more frequent (54.5% vs. 31.4%) than in the control group (without the umbilical cord around the fetal neck). The odds of a caesarean section were 2.62 (OR = 2.62) times greater in the study group. In the study group, the odds of umbilical cord wrapping during delivery were 2.57 times (OR = 2.57) greater than in the case of the fetuses in the control group.

**Figure 5:**
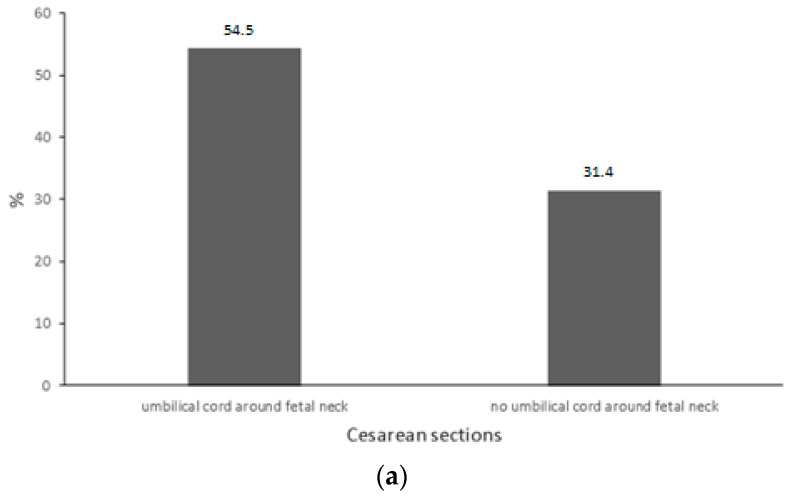

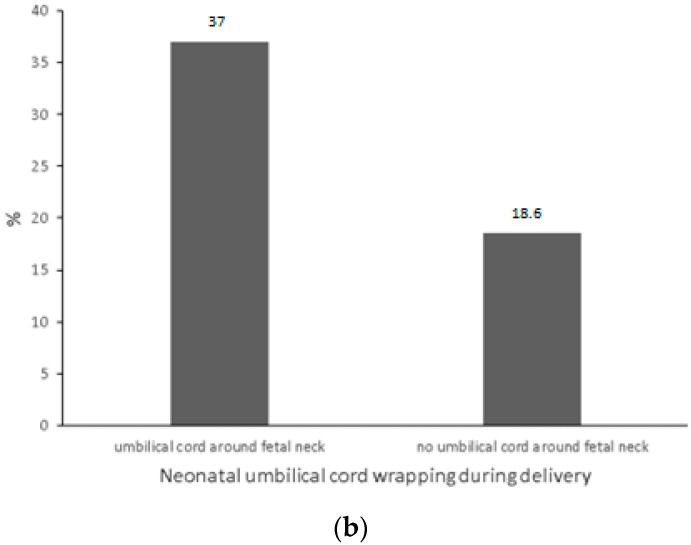
(**a**) Percentage distribution [%] of caesarean sections among fetuses in a study group (with the umbilical cord) and control group (without the umbilical cord around the fetal neck during echo-sonographic examination in the third trimester of pregnancy) (54.5% vs. 31.4%, *p* = 0.014). (**b**) Percentage distribution [%] of neonatal umbilical cord wrapping during delivery among fetuses from the study group (fetuses wrapped with the umbilical cord during echo-sonographic examination in the third trimester of pregnancy) and control group (37.0% vs. 18.6%, *p* = 0.027).

The percentage of newborns wrapped with the umbilical cord during delivery who were also wrapped with the umbilical cord during echo-sonographic examination in the third trimester of pregnancy was 37%, whereas in the case of fetuses who were not wrapped with the umbilical cord during the echo-sonographic examination but such wrapping was present during labor, the percentage of newborns was 18.6% (Table 2 and Figure 5a,b).

## 4. Discussion

The aim of our study was to answer the question of whether it is possible to measure the effect of the compressed umbilical cord on cardiac function and peripheral cord circulation in the third trimester in relation to obstetric and neonatal outcomes. This is primarily dictated by practical considerations, as the information about a fetus wrapped with the umbilical cord during an echo-sonographic examination may be associated with stress for the pregnant patient. The prognosis of the fetal condition, its circulatory efficiency, and perinatal observation are important issues in the context of possible adverse effects of the umbilical cord in the course of labor and in the postnatal condition of newborns [3,4].

The Tei index makes it possible to assess cardiac functionality globally and may be used for prenatal echocardiography [1,7]. The literature has devoted many publications to this echocardiographic parameter, drawing attention to its growth in the course of various fetal abnormalities, including congenital heart defects/congestive failure of fetal circulation or intrauterine growth restriction of the fetus [8,9]. The interest in the subject of fetal heart function during normal pregnancy is also increasing.

The Tei index for the RV was studied by Shi et al. [10] in fetuses wrapped in the umbilical cord, and it was higher in a group of 55 fetuses (23–40 weeks of gestation) in relation to the control group (0.42 ± 0.05 vs. 0.38 ± 0.05) (*p* < 0.05). For the LV, however, they found no significant differences between the study group and the control group [10].

Ghawi et al. [5] obtained mean fetal RV Tei values of 0.49 ± 0.09 for the study group with singleton healthy fetuses (250 cases) [5], while Chen et al. [11] elaborated fetal RV Tei normal ranges in 27.6 weeks of gestation (n = 18), and the mean value of the RV Tei was 0.39 ± 0.04 [11].

Ho et al. [12] analyzed fetal RV Tei in a group of 50 fetuses in the third trimester of pregnancy when mothers reported a reduction in fetal movements in relation to the control group (Tei RV 0.60 ± 0.12 vs. 0.59 ± 0.11) with no significant differences [12].

In our analysis, we did not find significantly higher RV Tei or LV Tei values in the study group compared to the control group (RV Tei 0.51 vs. 0.8; *p* = 0.335 and LV Tei 0.54 vs. 0.5; *p* = 0.099).

We made an attempt to determine the values of LV Tei and RV Tei on the basis of the ROC curve in both the study and control groups. However, the available data did not allow us to succeed.

The only differentiating parameter between these two groups was UMB PSV, which was higher for the study group (44.17 cm/s vs. 38.90 cm/s in the control group; *p* = 0.004). Very likely, the characteristic helical structure of the umbilical arterial vessels provides some form of adaptation for the alignment of velocity, pressure, temperature, shear strain rate, and static entropy within the arterial cycle.

The systolic pressure in the umbilical artery is about 25% lower compared to the umbilical vein [13,14]. Based on the studies on sheep, Troisi et al. [15] observed changes in the umbilical cord Doppler parameters in the fetuses of the sheep. They performed umbilical artery Doppler studies from 18 weeks of gestation, at weekly intervals, until delivery. The peak systolic velocity (PSV) in the umbilical artery and end-diastolic velocity (EDV) increased (*p* < 0.001), while the pulsatility index (PI) and resistance index (RI) decreased (*p* < 0.001) as pregnancy progressed. A linear trend was found for all analyzed Doppler parameters (*p* < 0.001) [15].

In our analysis, higher peak flow velocity parameters in the umbilical artery in the study group may indicate transient cord compression; however, it is not reflected in the fetal circulatory efficiency and has no significant effect on myocardial function, CTAR, or FHR.

When it comes to stillbirth, Quaresima et al. observed the umbilical cord around the stillborn baby’s neck in 28% of cases [16].

Our study has confirmed that all the monitored fetuses were born in good condition and on time. Maternal genitourinary tract infections did not significantly affect obstetric outcomes (details are presented in Table 1 and Table 2). The incidence of caesarean section was 2.62 times more frequent in the study group, while persistent ‘cord collision during labor’ was 2.57 times more frequent in the study group compared to the control group.

Despite these findings, we would recommend the addition of information on the presence or absence of umbilical cord wrapping around the fetal neck in medical records with an explanation regarding the lack of influence of this finding on fetal heart function.

Our study has very important clinical implications. It is a comprehensive description of prenatal echo-sonographic parameters in fetuses wrapped with the umbilical cord in the third trimester of pregnancy. Due to the fact that many patients ask and are worried about fetal and newborn outcomes when they get information about the fetus being wrapped with the umbilical cord around the fetal neck, our study may be a good source of knowledge for doctors and patients, as fetal umbilical cord wrapping in the third trimester of pregnancy does not have significant hemodynamic influence. However, the UMB PSV might be slightly elevated in this group, and the frequency of umbilical cord collisions during delivery and the need to perform a caesarean section at term seem to be more common.

The advantage of our study is that the fetal echocardiographic examinations were performed by an experienced specialist in echocardiography who analyzed in detail the course of the umbilical cord around the fetal neck. Moreover, it analyzes the follow-up examinations of newborns, which makes the study comprehensive.

In spite of the aforementioned advantages, the main limitation of our study is a small group of patients. Therefore, further studies must be focused on a larger group of patients and follow-up examinations of newborns with the inclusion of more echocardiographic and ultrasound parameters of the umbilical cord.

## 5. Conclusions

The presence of the umbilical cord around the fetal neck in the third trimester of pregnancy should not affect fetal cardiac function but may slightly increase the peak systolic velocity of the umbilical artery. However, in the analyzed group of fetuses, there was a higher rate of persistence of perinatal umbilical collision and the need to perform caesarean sections at term.

## Figures and Tables

**Table 1:** Comparison of fetuses in the study group (wrapped with umbilical cord during echo-sonography in the third trimester) (n = 46) and the control group (no umbilical cord) (n = 70). Bold shows (statistical significance of *p* < 0.05).

	Umbilical Cord Around the Fetal Neck (n = 46) Study Group		No Umbilical Cord Around the Fetal Neck (n = 70) Control Group		95% CI				
GA [Weeks] 28.1 ± 0.79 (M ± SD)	M	SD	M	SD	t	*p*	LL	UL	d Cohena
FHR [bpm]	147.67	11.00	149.04	10.01	0.69	0.491	–2.56	5.30	0.13
Tei RV	0.51	0.19	0.48	0.18	–0.97	0.335	–0.11	0.04	0.18
Tei LV	0.54	0.15	0.50	0.12	–1.66	0.099	–0.09	0.01	0.32
CTAR	0.30	0.04	0.28	0.04	–1.57	0.118	–0.03	0.00	0.31
UMBA PSV [cm/s]	44.17	10.09	38.90	8.10	–2.99	**0.004**	–8.77	–1.77	0.59
UMBA PI	1.15	0.31	1.09	0.25	–1.03	0.305	–0.16	0.05	0.20
AFI [cm]	16.88	3.65	16.68	4.01	–0.27	0.791	–1.66	1.27	0.05
EFW [g]	1579.02	881.68	1304.91	720.66	–1.83	0.070	–570.45	22.24	0.35
Week of delivery	39.24	1.32	39.06	1.52	–0.66	0.508	–0.73	0.36	0.13
Newborn weight [g]	3333.59	466.19	3422.29	589.95	0.86	0.393	–116.02	293.42	0.16
Apgar 1	9.83	0.68	9.84	0.44	0.16	0.872	–0.19	0.22	0.03
Apgar 5	9.93	0.33	9.99	0.12	1.01	0.315	–0.05	0.15	0.23
Hospital stay [days]	4.07	3.33	3.59	2.67	–0.86	0.393	–1.59	0.63	0.16

**Table 2:** Comparison of the fetuses with and without the umbilical cord around the fetal neck during echo-sonographic examination and analysis of variables. Bold shows (statistical significance of *p* < 0.05).

Umbilical Cord around the Fetal Neck During Prenatal Examination GA [Weeks] 28.1 ± 0.79 (M ± SD) and Follow-Up (Study Group)			No Umbilical Cord around the Fetal Neck During Prenatal Examination GA [Weeks] 28.1 ± 0.79 (M ± SD) and Follow-Up (Control Group)		*p*-Value of the Fisher’s Exact Test			
	n	%	n	%	χ^2^	*p*	φ	OR
Maternal urogenital tract infections	19	41.3	33	47.1	0.38	0.536	0.06	1.15
AGA	5	10.9	12	17.1	0.87	0.350	0.09	0.59
LGA	38	82.6	57	82.6	<0.01	1.000	<0.01	1.00
SGA	3	6.5	1	1.4		0.299	0.14	4.81
Spontaneous labour	22	55.0	48	69.6	2.34	0.126	0.15	0.54
Caesarean section	24	54.5	22	31.4	5.99	**0.014**	0.23	2.62
Umbilical cord around the newborn’s neck during delivery	17	37.0	13	18.6	4.89	**0.027**	0.21	2.57
Jaundice	14	31.8	22	31.4	<0.01	0.965	<0.01	1.02
Infections in newborns	7	15.6	9	13.2	0.12	0.729	0.03	1.21

## Data Availability

Not applicable.

## Abbreviations

PSV UMB	peak systolic velocity of umbilical artery
OR	odds ratio
NICU	neonatal intensive care unit
IVCT	isovolumetric contraction time
IVRT	isovolumetric relaxation time
ET	ejection time
UMB PI	umbilical artery pulsatility index
AFI	amniotic fluid index
EFW	estimated fetal weight
LGA	larger for gestational age
SGA	smaller for gestational age
AGA	appropriate for gestational age
RDS	neonatal respiratory distress syndrome
ROC	receiver operating characteristic curve
LV Tei	Tei index for the left ventricle
RV Tei	Tei index for the right ventricle
AUC	area under curve
EDV	end-diastolic velocity
PI	pulsatility index
RI	resistance index
CTAR	cardiothoracic area ratio
FHR	fetal heart rate
